# Nitrogen Fertilizer Factory Effects on the Amino Acid and Nitrogen Content in the Needles of Scots Pine

**DOI:** 10.1100/tsw.2001.386

**Published:** 2001-12-04

**Authors:** Eugenija Kupsinskiene

**Affiliations:** Department of Botany, University of Agriculture of Lithuania, Kaunas-Akademija, Lithuania

**Keywords:** Pinus sylvestris, conifers, arginine, free amino acids, protein, element concentration, industrial pollution

## Abstract

The aim of the research was to evaluate the content of amino acids in the needles of Pinus sylvestris growing in the area affected by a nitrogen fertilizer factory and to compare them with other parameters of needles, trees, and sites. Three young-age stands of Scots pine were selected at a distance of 0.5 km, 5 km, and 17 km from the factory. Examination of the current-year needles in winter of the year 2000 revealed significant (p

Eugenija Kupcinskiene

**The aim of the research was to evaluate the content of amino acids in the needles of *Pinus sylvestris* growing in the area affected by a nitrogen fertilizer factory and to compare them with other parameters of needles, trees, and sites. Three young-age stands of Scots pine were selected at a distance of 0.5 km, 5 km, and 17 km from the factory. Examination of the current-year needles in winter of the year 2000 revealed significant ($p < 0.05$) differences between the site at a 0.5-km distance from the factory and the site at a 17-km distance from the factory — with the site closest to the factory showing the highest concentrations of protein (119%), total arginine (166%), total other amino acids (depending on amino acid, the effect ranged between 119 and 149%), free arginine (771%), other free amino acids (glutamic acid, threonine, serine, lysine — depending on amino acid, the effect ranged between 162 and 234%), also the longest needles, widest diameter, largest surface area, and heaviest dry weight (respectively, 133, 110, 136, and 169%). The gradient of nitrogen concentration in the needles was assessed on the selected plots over the period of 1995–2000, with the highest concentration (depending on year, 119 to 153%) documented in the site located 0.5 km from the factory. Significant correlations were determined between the total amino acid contents (r = 0.448 – 0.939, $p < 0.05$), some free amino acid (arginine, aspartic acid, glutamic acid, lysine, threonine, and serine) contents (r = 0.418 – 0.975, $p < 0.05$), and air pollutant concentration at the sites, the distance between the sites and the factory, and characteristics of the needles. No correlation was found between free or total arginine content and defoliation or retention of the needles. In conclusion, it was revealed that elevated mean monthly concentration of ammonia (26 µg $m^{-3}$) near the nitrogen fertilizer factory caused changes in nitrogen metabolism, especially increasing (nearly eight times) concentration of free arginine in the needles of Scots pine.**

## INTRODUCTION

Evidence that enhanced atmospheric nitrogen is affecting semi-natural terrestrial ecosystems comes from historic and present-day research[1,2,3,4]. Wide scale damages to *Pinus sylvestris* under ammonia pollution have been already described: the excess of nitrogen causes ultrastructural changes[5], disturbs the nutrition balance[6,7], and may affect the whole ecosystem, changing competitive ability and physiological stability[2,3] of the species. In the 1980s in some forests of West, Central, and Eastern Europe and North America, the deposition of nitrogen was higher than the estimated amount of the element that could enter biomass[8,9].

It has been shown that, in the presence of high ammonia concentration, trees absorb more ammonia than the amount that can be utilized for protein synthesis; therefore nitrogen accumulates in the form of the arginine[10,11], the amount of which in the needles may increase by several up to a hundred times[1,7,9,10,12]. The alterations in N flux affect the arginine content in the needles within an interval[l3,14] of several hours. Elevated amounts of other free amino acids and polyamines were also found to be a consequence of atmospheric ammonia excess[15,16,17]. In most cases, changes in the content of free amino acids are documented during the experiments on plants that grow under partially controlled/limited nitrogen additions[19,20] and, in a few cases, in the polluted natural environment[19,20]. Furthermore, field experiments very often are done in areas situated close to large cattle-breeding farms[21], and little information is available on the effects of industrial or urban-origin ammonia[1].

Since the 1970s the nitrogen fertilizer plant has been among the main industrial pollution sources of Lithuania, annually emitting 15,000 to 40,000 t of pollutants and causing a deposition of more than 120 kg N $ha^{-1}$ $year^{-1}$ near the factory in 1989. In the last decade the area affected by the factory became a subject of intensive air, soil[22], general tree condition[23], and latent injury observations[24,25,26,27]. At present, due to changes in technologies, pollutant emissions have decreased several times, though pollution effects are still detectable[24,25].

The aim of the research was to determine the amount of amino acids in the needles of *P. sylvestris*, sampled from the trees growing at different distances from the nitrogen fertilizer plant in Lithuania, and to compare the obtained data with the other parameters of the needles, trees, and characteristics of the sites.

## METHODS

For the investigation of the content of amino acids of *P. sylvestris* needles, samples of needles were collected in the area affected by the nitrogen fertilizer plant *Achema* (*Azotas*), which released over 6000 t of pollutants into the atmosphere through the year 1999, including 196 t of ammonia and 1611 t of nitrogen oxides (data from the Ministry of Environment of the Republic of Lithuania). Three sites with young-age *Vaccinio-myrtillo-Pinetum* stands were selected for the study: 0.5 km west of the factory, and 5 km and 17 km northeast of the factory (Table 1). Analysis of the mean monthly concentrations of $NO_2$ and $NH_3$, visual evaluation of the trees, and needle sampling were done as they were described earlier[27,28].

December of the year 2000 was chosen for collecting the samples of the needles, in order to avoid rapid fluctuations in concentrations of the nutrient and dry mass, also as a month of the season when the highest concentrations of arginine might be found[19,28].

For the analysis of the total amount of amino acids, lipids were removed using diethyl ether (16 h, 36°C); then 0.15 g of the sample was hydrolysed with 6 *N* hydrochloric acid (for 24 h at 104°C)[29]. The amount of proteins and lipids was determined in four trees of each site. The hydrolysate was cooled and filtered. The hydrochloric acid was removed from hydrolysate using a vacuum rotary evaporator (40°C). The dry precipitate was dissolved in sodium citrate–hydrogen chloride buffer solution (pH 2.20), and the filtrate was kept for the analysis in the cold (–20°C). The amount of amino acids was determined with an amino acid analyzer T 339 (Mikrotechna, Czech Republic) using Ostion LG ANB ion-exchange resin (Lachema, Czech Republic). For the elution of amino acids, 3 buffer solutions were used: sodium citrate–hydrogen chloride–sodium chloride (0.30 *N* sodium, pH 3.5 and 0.40 *N* sodium, pH 4.25) and boric acid–sodium hydroxide (0.45 *N* sodium, pH 9.45). The rate of the flow of the buffer solutions through the column was 26 ml $h^{-1}$; the rate of the flow of the ninhydrin was 14 ml $h^{-1}$. The injection volume of the samples was 400 ml. The separation of amino acids occurred at the temperature of 58 and 70°C, and their concentrations were determined at a wavelength of 520 nm. For data analysis, a TZ 4100 recorder (Laboratorni Pristroje, Czech Republic) and a Digital Integrator (Model 26 C, Analitik, Bulgaria) were used. The duration of the full cycle of analysis (including the regeneration and stabilization of the column) was 120 min. For the column regeneration, a solution of 0.2 *N* sodium hydroxide was used. The amino acids produced by the Lachema (Czech Republic) were used as standards.

For the determination of the amount of free amino acids, 4 to 6 g of fresh needle mass was homogenized for 10 min in the cold in 30 ml of 4% sulfosalicylic acid with 0.1 ml 25 µmol $ml^{-1}$ norleucine (internal standard); later the mass was filtered, and the filtrate was adjusted to pH 2.2 with 1 *N* sodium hydrox-

**TABLE 1**
**Characteristics (Mean Values) of Sampled Trees and Sites Near the Factory[27]**

| Parameters | 1 Site (0.5 km) | 2 Site (5 km) | 3 Site (17 km) |
|---|---|---|---|
| $NH_3$, µg $m^{-3}$ | 14.8 | 6.6 | 4.0 |
| $NO_2$, µg $m^{-3}$ | 6.1 | 4.2 | 3.4 |
| Age of trees, year | 24 | 30 | 27 |
| Height of trees, m | 11.5 | 10.4 | 12.0 |
| Diameter of tree, cm | 20.5 | 19.3 | 17.0 |
| Defoliation, % | 11.0 | 22.5 | 12.5 |
| Needle retention, year | 2.8 | 2.1 | 3.2 |

ide. The subsequent determination was the same as described for the total amount of amino acids. The amount of protein was determined by Kjeldahl method[30].

Part of the needle material collected in the field was left for the estimation of morphological and gravimetric parameters and total N amount. The dry weight (d.w. in g for 100 needles), length (mm), diameter (mm), surface area ($mm^2$)[31], and specific leaf area (SLA, $mm^2$ $g^{-1}$) of the needles were determined. In each site, data for four trees (in each tree, 100 needles from the same branch were sampled, and mean value was calculated) were used for evaluation of morphological parameters. In 1995–1998, samplings for N analyses were done in September. The needles were homogenized by a Mühle (Germany) mill (with a 0.75-mm density sieve), and the amount of N was determined by a NIR analyser PSCO/ISI IBM-PC 4250 (Pacific Scientific, USA)[24]. Dispersion and correlative analysis were used for statistical evaluation of the data (Minitab 10.5; SPSS 8). The results for the amino acids and nitrogen are shown as the averages of eight measurements taken from eight trees as replicates.

## RESULTS

Comparing the three sites situated within 0.5- to 17-km distance from the factory, significant differences ($p < 0.05$) were found in the amount of proteins in the needles — at the site 17 km away from the factory, they had the smallest amount of proteins; whereas the protein content of the needles collected from the closer areas was higher (112 to 119%; see Table 2). No significant differences among the sites were found, according to the amount of lipids.

While estimating the total amounts of individual amino acids (Table 3), with the exception of proline, significant differences ($p < 0.05$) were found between the needles collected from the site most distant from the factory (100%) and sites closer to the plant (0.5 and 5 km, respectively), where higher contents of arginine (166 and 137%), aspartic acid (149 and 130%), lysine (139 and 136%), glutamic acid, threonine, tyrosine, phenylalanine, histidine, glycine (138 and 121%), and smaller increases of the other amino acids (129 and 114%) were estimated. The needles of Scots pines growing 0.5 km away from the factory were significantly different in the amount of most amino acids (except proline, methionine, and lysine) from the needles collected at the site located at a distance of 5 km from the plant ($p < 0.05$).

The comparison of the amount of separate free amino acids in the needles collected at the three sites revealed significant differences ($p < 0.05$) only in the amount of arginine (771 and 279%), glutamic acid (154 and 140%), threonine (234 and 134%), serine (231 and 131%), and lysine (230% at 0.5 km) content determined in the needles collected at the sites 0.5 and 5.0 km away from the factory, as compared with 100% assessed at the site 17 km away from the plant (see Table 4). Comparison of different amino acids in the needles at the sites has shown that the concentration gradient was the highest both in the case of total and free arginine (respectively, 1.7 and 7.7 times higher close to the factory). Needles from the site 0.5 km away from the factory showed the most pronounced changes in the amount of free amino acids (Table 4), while the site with the most contrast in the total amount of the amino acids in the needles (Table 5) was at a distance of 17 km from the plant.

Over the 1995–2000 period, the needles collected at a 0.5-km distance from the factory had the highest amount of nitrogen (119–153%); at the site 5 km from the plant, the concentration was intermediate (96–113%) when compared with the amount (100%, Table 5) assessed in the needles collected at the site 17 km away from the factory.

According to the morphological parameters, the most significantly different site was 17 km away from the factory. The length, diameter, surface area, and dry weight of the needles there were, respectively, 1.33, 1.1, 1.36, and 1.69 times smaller — whereas SLA was 1.26 times higher than at the site 0.5 km away from the plant (Table 5). The driest site (d.w./f.w) was 5 km away from the factory, where the trees were more defoliated ($p < 0.01$) than at the other sites (Tables 1, 6).

Significant correlations (r = 0.669–0.939, $p < 0.05$) were found between the total amount of individual amino acids in the needles and the distance of the investigation sites from the factory, concentrations of $NH_3$, $NO_2$, morphological parameters, dry weight, SLA, protein and nitrogen amounts of the needles; whereas the relation with the defoliation was not detected. Correlations between total proline or methionine amount with the above-mentioned parameters were lower (r = 0.448 – 0.595).

The significant correlations between the amount of free amino acids, the parameters of the needles, and site characteristics were documented only for a few amino acids – arginine, as-

**TABLE 2**
**Amount of Proteins and Lipids (mg $g^{-1}$ d.w.) in Current-Year Needles of *P. sylvestris* Growing in the Area Affected by the Nitrogen Fertilizer Factory (December 2000)**

| | 1 Site (0.5 km) | | 2 Site (5 km) | | 3 Site (17 km) | | |
|---|---|---|---|---|---|---|---|
| Compound | M | SD | M | SD | M | SD | Significant Differences[a] |
| Proteins | 98 | 3 | 94 | 2 | 84 | 2 | 1,2–3 |
| Lipids | 107 | 2 | 117 | 3 | 105 | 3 | n.s. |

*Note:* M = mean; SD = standard deviation.

[a] Significant differences between sites ($p < 0.05$); 1 = 1 site; 2 = 2 site; 3 = 3 site; n.s. = insignificant difference.

**TABLE 3**
**Content of Total Amino Acids (μmol g$^{-1}$ d.w.) in Current-Year Needles of *P. sylvestris* Growing in the Area Affected by the Nitrogen Fertilizer Factory (December 2000)**

| Amino Acid | 1 Site (0.5 km) M | 1 Site (0.5 km) SD | 2 Site (5 km) M | 2 Site (5 km) SD | 3 Site (17 km) M | 3 Site (17 km) SD | Significant Differences[a] |
|---|---|---|---|---|---|---|---|
| ALA | 59.4 | 3.3 | 56.7 | 1.5 | 45.7 | 3.4 | 1,2–3; 1–2 |
| ARG | 31.6 | 2.2 | 26.1 | 1.4 | 19.0 | 2.9 | 1,2–3; 1–2 |
| ASP | 81.1 | 7.6 | 70.8 | 7.8 | 54.4 | 2.5 | 1,2–3; 1–2 |
| GLN | 85.2 | 5.6 | 79.5 | 4.4 | 64.5 | 3.0 | 1,2–3; 1–2 |
| GLY | 78.7 | 6.4 | 71.6 | 4.7 | 58.0 | 2.9 | 1,2–3; 1–2 |
| HIS | 21.3 | 0.9 | 19.8 | 5.1 | 15.5 | 1.6 | 1,2–3; 1–2 |
| ILE | 21.9 | 1.2 | 20.5 | 7.4 | 18.1 | 4.1 | 1,2–3; 1–2 |
| LEU | 55.0 | 2.6 | 51.8 | 1.3 | 43.8 | 1.0 | 1,2–3; 1–2 |
| LYS | 27.0 | 1.4 | 26.2 | 1.2 | 19.4 | 2.4 | 1,2–3 |
| MET | 7.6 | 1.0 | 6.7 | 1.0 | 5.9 | 0.1 | 1–3 |
| PHE | 33.7 | 1.3 | 30.8 | 4.1 | 25.4 | 1.1 | 1,2–3; 1–2 |
| PRO | 35.9 | 3.5 | 35.5 | 5.0 | 30.1 | 5.4 | n.s. |
| SER | 40.5 | 2.3 | 38.1 | 1.4 | 31.8 | 1.2 | 1,2–3; 1–2 |
| THR | 35.1 | 1.7 | 32.2 | 1.7 | 26.1 | 2.6 | 1,2–3; 1–2 |
| TYR | 18.6 | 0.9 | 17.5 | 5.4 | 14.0 | 1.2 | 1,2–3; 1–2 |
| VAL | 35.9 | 2.4 | 33.7 | 1.1 | 28.1 | 1.8 | 1,2–3; 1–2 |

*Note:* M = mean; SD = standard deviation.
Abbreviations of amino acids: ALA = alanine, ARG = arginine, ASP = aspartic acid, GLU = glutamic acid, GLY = glycine, HIS = histidine, ILE = isoleucine, LEU = leucine, LYS = lysine, MET = methionine, PHE = phenylalanine, PRO = proline, SER = serine, THR = threonine, TYR = tyrosine, VAL = valine.

[a] Significant differences between sites ($p < 0.05$); 1 = 1 site; 2 = 2 site; 3 = 3 site.

partic acid, glutamic acid, lysine, serine, and threonine (Table 7). A few statistically significant correlations were found between the studied amino acid contents and the parameters of the trees.

## DISCUSSION

In the area that was selected for the research, the sites differed by a factor of 6.5 in the average monthly concentrations of ammonia and a factor of 1.8 in the amount of nitrogen dioxide[27]. Within the distance of 0.5 km from the factory, mean monthly concentration of ammonia (26 μg m$^{-3}$) exceeded the critical monthly concentrations (23 μg m$^{-3}$) and was higher than critical annual ammonia concentrations (8 μg m$^{-3}$). The highest mean monthly ammonia concentrations detected near the factory were 45.2–69.3 μg m$^{-3}$. The lowest $NH_3$ and $NO_2$ concentrations (1.0–1.8 μg m$^{-3}$ and 1.9–2.2 μg m$^{-3}$, respectively) were similar to the European background concentrations of unpolluted areas[1]. In 1996, the nitrogen deposition in the area under the effect of the factory made 17–48 kg ha$^{-1}$ year$^{-1}$ gradient[22].

Contradictory data exist concerning relations of the arginine content of the needles and the damage[19] to the tree. In our study, visual evaluation of the trees has shown that the highest defoliation was assessed at the site 5 km away from the factory — in the area which was neither distinguished by the highest concentration of ammonia in the air nor by the highest concentration of the free amino acids or nitrogen in the needles of Scots pine. Other pollution factors, like the hydrotop of the habitat, might influence the state of the trees[32].

The higher amounts of the free arginine, glutamic acid, lysine, serine, threonine, and the amount of proteins detected next to the factory support the idea that, in the presence of elevated concentrations of ammonia, the amount of organic nitrogen can increase greatly in the plants[1,3,12]. Acute nitrogen-induced changes have been found to exist only in the vicinity of sources with extremely high nitrogen emissions[4]. The data of our study support that finding. The nearly eight-times higher amount of the free arginine estimated in the needles collected near the factory decreases, at the 5-km site, to 1.4 times when compared with the site 17 km away from the factory. Epicuticular wax observations on the surface of the needles revealed higher erosion only in the site 0.5 km away from the factory[25]. Our investigation shows that pollution is significantly expressed, but is not very high when compared with effects described in the literature that reach 20- to 100-fold increases[9,12] in arginine content.

**TABLE 4**
**Content of Free Amino Acids (μmol $g^{-1}$ d.w.) in Current-Year Needles of *P. sylvestris* Growing in the Area Affected by the Nitrogen Fertilizer Factory (December 2000)**

| | 1 Site (0.5 km) | | 2 Site (5 km) | | 3 Site (17 km) | | |
|---|---|---|---|---|---|---|---|
| **Amino acid** | **M** | **SD** | **M** | **SD** | **M** | **SD** | **Significant Differences[a]** |
| ALA | 1.02 | 0.18 | 1.00 | 0.14 | 0.88 | 0.15 | n.s. |
| ARG | 3.61 | 0.46 | 1.31 | 0.06 | 0.47 | 0.02 | 1–2,3 |
| ASP | 3.33 | 0.17 | 3.03 | 0.29 | 3.15 | 0.13 | n.s. |
| GLN | 3.01 | 0.21 | 2.74 | 0.18 | 1.95 | 0.13 | 1–2, 3 |
| GLY | 0.17 | 0.02 | 0.14 | 0.43 | 0.14 | 0.07 | n.s. |
| HIS | 0.82 | 0.09 | 0.78 | 0.17 | 0.79 | 0.05 | n.s. |
| ILE | 0.16 | 0.04 | 0.14 | 0.02 | 0.14 | 0.21 | n.s. |
| LEU | 0.14 | 0.02 | 0.12 | 0.02 | 0.12 | 0.15 | n.s. |
| LYS | 0.46 | 0.02 | 0.21 | 0.04 | 0.20 | 0.03 | 1–2, 3 |
| PHE | 1.12 | 0.17 | 1.16 | 0.21 | 1.08 | 0.15 | n.s. |
| PRO | 1.65 | 0.25 | 1.42 | 0.33 | 1.40 | 0.50 | n.s. |
| SER | 1.04 | 0.29 | 0.59 | 0.14 | 0.45 | 0.06 | 1–2, 3 |
| THR | 0.89 | 0.25 | 0.51 | 0.12 | 0.38 | 0.05 | 1–2, 3 |
| TYR | 0.71 | 0.01 | 0.76 | 0.01 | 0.76 | 0.10 | n.s. |
| VAL | 0.59 | 0.05 | 0.65 | 0.10 | 0.59 | 0.03 | n.s. |

*Note:* M = mean; SD = standard deviation.
Abbreviations of amino acids: ALA = alanine, ARG = arginine, ASP = aspartic acid, GLU = glutamic acid, GLY = glycine, HIS = histidine, ILE = isoleucine, LEU = leucine, LYS = lysine, MET = methionine, PHE = phenylalanine, PRO = proline, SER = serine, THR = threonine, TYR = tyrosine, VAL = valine.

[a] Significant differences between sites ($p < 0.05$); 1 = 1 site; 2 = 2 site; 3 = 3 site; n.s. = insignificant difference.

**TABLE 5**
**Concentration of Nitrogen (mg $g^{-1}$ d.w.) in Current-Year Needles of *P. sylvestris* Growing in the Area Affected by the Nitrogen Fertilizer Factory**

| | 1 Site (0.5 km) | | 2 Site (5 km) | | 3 Site (17 km) | | |
|---|---|---|---|---|---|---|---|
| **Time of Sampling** | **M** | **SD** | **M** | **SD** | **M** | **SD** | **Significant Differences[a]** |
| September, 1995 | 20.3 | 1.7 | 16.4 | 1.0 | 17.0 | 1.2 | 1–2,3 |
| September, 1996 | 18.1 | 2.1 | 13.2 | 2.3 | 11.8 | 1.0 | 1–2,3 |
| September, 1997 | 17.7 | 1.4 | 13.9 | 1.1 | 13.7 | 1.7 | 1–2,3 |
| September, 1998 | 20.1 | 2.0 | 15.6 | 1.7 | 15.1 | 2.1 | 1–2,3 |
| December, 2000 | 20.7 | 4.2 | 18.7 | 3.4 | 16.6 | 7.0 | 1–2,3 |

*Note:* M = mean; SD = standard deviation.

[a] Significant differences between sites ($p < 0.05$); 1 = 1 site; 2 = 2 site; 3 = 3 site.

The biochemical differences we detected between the sites were similar in protein amount and smaller in nitrogen and free amino acid contents, when compared with the data of the study performed on *P. sylvestris* seedlings, grown in four- to six-times higher atmospheric concentrations of ammonia[12].

**TABLE 6**
**Morphological and Gravimetric Parameters of Current-Year Needles of *P. sylvestris* Growing in the Area Affected by the Nitrogen Fertilizer Factory (December 2000)**

| Parameters of the Needles | 1 Site (0.5 km) | | 2 Site (5 km) | | 3 Site (17 km) | | Significant Differences[a] |
|---|---|---|---|---|---|---|---|
| | M | SD | M | SD | M | SD | |
| Diameter (mm) | 0.75 | 0.01 | 0.76 | 0.02 | 0.68 | 0.04 | 1,2–3 |
| Length (mm) | 80 | 1 | 65 | 4 | 59.7 | 6.0 | 1–3 |
| Area ($mm^2$) | 323 | 5 | 260 | 18 | 227 | 26 | 1–3 |
| d.w./f.w. (%) | 38.9 | 0.3 | 40.0 | 0.2 | 28.2 | 0.6 | 2–1,3 |
| Dry weight (g) | 3.15 | 0.20 | 2.47 | 0.29 | 1.86 | 0.42 | 1–3 |
| SLA ($mm^2\ g^{-1}$) | 102.7 | 5 | 105.6 | 5.2 | 129.5 | 12 | 1,2–3 |

*Note:* M = mean; SD = standard deviation.

[a] Significant differences between sites ($p < 0.05$); 1 = 1 site; 2 = 2 site; 3 = 3 site.

**TABLE 7**

**Correlation Coefficients between Site, Tree, Needle Characteristics and Content of Free Amino Acids in Current-Year Needles of *P. sylvestris* Growing in the Area Affected by the Nitrogen Fertilizer Factory**

| | ARG | ASP | GLU | LYS | SER | THR |
|---|---|---|---|---|---|---|
| Distance of site | 0.865** | n.s. | 0.941** | 0.687** | 0.721** | 0.730** |
| $NH_3$ | 0.975** | 0.451* | 0.742** | 0.868** | 0.799** | 0.811** |
| $NO_2$ | 0.963** | n.s. | 0.856** | 0.827** | 0.799** | 0.810** |
| Defoliation | n.s. | –0.459* | n.s. | –0.494* | n.s. | n.s. |
| Retention of needles | n.s. | n.s. | n.s. | n.s. | n.s. | n.s. |
| Length of needles | 0.974** | 0.418* | 0.801** | 0.854** | 0.805** | 0.815** |
| Diameter of needles | 0.581** | n.s. | 0.885** | n.s. | 0.487* | 0.494* |
| Area of needles | 0.973** | 0.412* | 0.811** | 0.850** | 0.805** | 0.815** |
| Weight of needles | 0.937** | n.s. | 0.901** | 0.784** | 0.789** | 0.790** |
| SLA | –0.737** | n.s. | –0.936** | –0.538** | –0.616** | –0.622** |
| Proteins | 0.836** | n.s. | 0.945** | 0.652** | 0.688** | 0.706** |
| N (2000 year) | 0.923** | n.s. | 0.915** | 0.763** | –0.770** | 0.778** |

*Note:* n.s.= insignificant correlations; significance of correlation: * $p < 0.05$; ** $p < 0.01$. Abbreviations of amino acids: ARG = arginine, ASP = aspartic acid, GLU = glutamic acid, LYS = lysine, SER = serine, THR = threonine.

Concentrations of nitrogen in the current-year needles sampled at a 0.5-km distance from the factory were much higher than in other forest areas of Lithuania[33,34], and in 4 out of 5 years they belonged to the range defined as an ecological excess or physiological optimum. In the other sites, in most cases, N concentration was considered as normal or belonging to an ecological optimum content[1,35]. Very similar concentrations of nitrogen in the 1995- and 2000-year studies were in agreement with small variations in the factory emissions at that time and did not show any decreasing or increasing tendency over 5 years estimation, though in the years 1965–1990 the area near the factory suffered from the heavy pollution reaching up to 40,000 t year$^{-1}$[22]. The concentration of nitrogen in the needles taken from the selected sites varied within the 5-year period of investigation, being the lowest in the year 1997[24]. The fact of annual fluctuation of N concentration in the needles is also described in other studies[36]. It is documented that the concentration of nitrogen changes throughout the vegetation period and is much higher in the late autumn than in the summer[19]. In our study, the amounts of N in the needles sampled in September 1998 and

December 2000 were very similar. The selected sites were distinguished by similar, nitrogen-poor, sandy soils; therefore the detected differences in nitrogen concentration reflect the gradient of pollutants. The proportion between the amount of arginine and the corresponding concentration of nitrogen in the needles was similar to that described by Schmeink and Wild[19] and it was much lower than the data reported by Huhn and Schultz[9] or Nasholm et al.[37].

In some reports, changes in arginine amount were documented in relation to nutrient deficiency in the needles[17,29,38]. Neither macro nor micronutrient deficiency was found at the sites of the present study during 1997 estimations, nor were significant differences in element concentrations among the sites[24] revealed.

A xeromorphic structure is typical of the needles growing in natural habitats. The excess of nitrogen in the current-year needles causes changes that are directed towards the mesomorphic changes of the xeromorphic structure: the length and diameter of the needles increases[5]. That is reflected in our results as well: the length, diameter, and surface area of the needles that contain more nitrogen and proteins are higher. The smaller specific leaf area of the needles collected at the site close to the factory shows that the photosynthesis of the needles in that plot is more effective.

The metabolism of the *P. sylvestris* adapts to concentrations of ammonia that exceed the average concentrations three to ten times[12]. The morphophysiological parameters documented in our research show that growth of the needles is the best at the site next to the nitrogen fertilizer factory.

## CONCLUSIONS

1. Comparing the young stands of *P. sylvestris* within 0.5- to 17-km distance from the nitrogen fertilizer factory (*Achema*), distinguished by 26.0–4.0 µg $m^{-3}$ $NH_3$ and 6.1–3.4 µg $m^{-3}$ $NO_2$ gradients, it was observed that, at the site 0.5 km away from the factory, the following needles parameters were the highest ($p < 0.05$): the amount of proteins (119%), the amount of total arginine (166%), total aspartic acid (149%), total amount of other amino acids (139–114%), the amount of free arginine (771%), free glutamic acid (162%), free threonine (232%), free serine (233%), free lysine (234%), the amount of nitrogen (125%), and the length, diameter, surface area, and dry mass of the needles (133, 110, 136, and 169%, respectively).
2. Significant correlations ($p < 0.05$) were found between the total amount of separate amino acids and the parameters of the needles and sites. The significant correlations of the amount of free amino acids with the characteristics of the needles and the sites were found only for some amino acids – arginine, glutamic acid, aspartic acid, lysine, threonine, and serine.
3. It is possible to conclude that the increased concentration of pollutants in the air close to the factory, and the concentration of atmospheric ammonia in particular, cause the changes in the conifer nitrogen cycle, with the particular increase (nearly eight times) in the amount of free arginine.

## ACKNOWLEDGEMENTS

The work was funded by the Joint Stock Company *Achema*. Author thanks to Professor Satu Huttunen, Dr. Saulius Bliznikas and Almantas Kliucius for advice and assistance in the research. The English of the manuscript was revised by Mr. Petras Simaska.

| | | |
|---|---|---|
| Received: | July | 13, 2001 |
| Revised: | November | 11, 2001 |
| Accepted: | November | 12, 2001 |
| Published: | December | 4, 2001 |
